# The Response of Net Primary Production to Climate Change: A Case Study in the 400 mm Annual Precipitation Fluctuation Zone in China

**DOI:** 10.3390/ijerph16091497

**Published:** 2019-04-27

**Authors:** Yang Li, Yaochen Qin

**Affiliations:** 1 Key Laboratory of Geospatial Technology for Middle and Lower Yellow River Regions, College of Environment and Planning, Henan University, Kaifeng 475004, China; liyanghenu@163.com; 2 Henan Collaborative Innovation Center of Urban-Rural Coordinated Development, Zhengzhou 450046, China

**Keywords:** climate change, 400 mm annual precipitation, net primary production, spatial and temporal variation, second-order partial correlation, Hurst index

## Abstract

The regions in China that intersect the 400 mm annual precipitation line are especially ecologically sensitive and extremely vulnerable to anthropogenic activities. However, in the context of climate change, the response of vegetation Net Primary Production (NPP) in this region has not been scientifically studied in depth. NPP suffers from the comprehensive effect of multiple climatic factors, and how to eliminate the effect of interfering variables in the correlation analysis of NPP and target variables (temperature or precipitation) is the major challenge in the study of NPP influencing factors. The correlation coefficient between NPP and target variable was calculated by ignoring other variables that also had a large impact on NPP. This increased the uncertainty of research results. Therefore, in this study, the second-order partial correlation analysis method was used to analyze the correlation between NPP and target variables by controlling other variables. This can effectively decrease the uncertainty of analysis results. In this paper, the univariate linear regression, coefficient of variation, and Hurst index estimation were used to study the spatial and temporal variations in NPP and analyze whether the NPP seasonal and annual variability will persist into the future. The results show the following: (i) The spatial distribution of NPP correlated with precipitation and had a gradually decreasing trend from southeast to northwest. From 2000 to 2015, the NPP in the study area had a general upward trend, with a small variation in its range. (ii) Areas with negative partial correlation coefficients between NPP and precipitation are consistent with the areas with more abundant water resources. The partial correlation coefficient between the NPP and the Land Surface Temperature (LST) was positive for 52.64% of the total study area. Finally, the prediction of the persistence of NPP variation into the future showed significant differences on varying time scales. On an annual scale, NPP was predicted to persist for 46% of the study area. On a seasonal scale, NPP in autumn was predicted to account for 49.92%, followed by spring (25.67%), summer (13.40%), and winter (6.75%).

## 1. Introduction

Net primary production (NPP) refers to the total amount of organic matter accumulated by vegetation per unit time and area. It is the difference between gross primary productivity (GPP) and autotrophic respiration (AR) produced through plant photosynthesis [1]. As an important part of the terrestrial ecosystem, vegetation can effectively connect and exchange energy between the atmosphere, water, and soil [2]. NPP results from the interaction between plants, and the environment represents the productivity of plants and the response of the ecosystem to climate change. Simultaneously, food and fuel for human survival and development come from NPP, an essential indicator of the sustainability of terrestrial ecosystems [3]. Therefore, it is of great significance to effectively evaluate the current situation and predict future trends of NPP. This study is valuable for understanding the impact of climate change on the environment and the response mechanisms of vegetation to climate change.

Theoretical studies of NPP have mainly focused on the improvement of estimation methods [4,5,6,7]. On the other hand, empirical studies have primarily focused on the spatial and temporal patterns and changes in NPP [8,9,10,11], the factors that affect these changes [12,13,14,15,16], the responses to climate change [14,17], and predictions of future trends [18]. Wang et al. [19] studied the spatial–temporal variations and factors that affect the NPP of vegetation and concluded that NPP is significantly affected by rainfall and the increase in urbanization. Gao et al. [20] found that during the 1980s and 1990s, the decrease in precipitation decreased the NPP in the transition zone between agriculture and livestock farming in China. Zhao et al. [21] found a significant correlation between NPP and evapotranspiration by studying the degree to which the drought in southwestern China affected NPP processes. Previous studies have used simple correlation analysis to study the impact of climate change on NPP, and such studies have confirmed that NPP is simultaneously affected by a variety of climate factors and human activities [22]. As a result, it is difficult to adequately quantify the impact of a single climate factor on NPP. Vast amounts of literature focusing on the impact of climate change on NPP were reviewed as part of this study, and three critical research gaps were revealed. First, previous studies have considered annual precipitation data from 824 meteorological stations in mainland China. In contrast, this study incorporates the analysis and spatial interpolation of data from over 2400 meteorological stations. Second, most previous studies that aimed to predict the persistence of NPP have been undertaken on an annual scale and rarely examined the more detailed seasonal scale. Furthermore, limited methodologies have been used in these studies for determining the current patterns, variation trends, influence of climate change, and the persistence of NPP into the future in the 400 mm Annual Precipitation Fluctuation Zone. The influence of climate factors on vegetation NPP has been previously analyzed by a simple correlation analysis method without excluding other variables closely related to the target, and such omissions lead to errors. In addition, many recent studies have been reported on contemporary real-life case studies of machine learning techniques in hydrologic prediction. Moazenzadeh et al. [23] attempted to solve the problem that most meteorological stations in developing countries do not record evaporation. A new hybrid algorithm was used to predict evaporation, and the performance of support vector regression (SVR) and SVR model coupled with firefly algorithm (SVR-FA) model in predicting the daily evaporation of Lahijan and Rasht stations in northern Iran was evaluated. Chen et al. [24] presented a three-person multi-objective conflict decision model for reservoir flood control. This model is simple and easy to operate and can be applied to practical problems. It has been verified in the practical application of Fengman reservoir in China. Yaseen et al. [25] verified the feasibility of enhanced version of extreme learning machine (EELM) model in river flow prediction in tropical environments. In the context of climate change, the increased frequency of extreme flooding poses a severe threat to economy and human security. Therefore, it is necessary to accurately predict the time and magnitude of peak discharge before the arrival of flood. Chau [26] proposed that meta-heuristic techniques can be used to calibrate data-driven rainfall and runoff models to improve the prediction accuracy. In view of the lagged prediction effect that often occurs during neural network modeling, Wu and Chaw [27] attempted to eliminate the lag effect from modular artificial neural network (MANN) and data preprocessing by singular spectrum analysis (SSA). Ghorbani et al. [28] adopted the particle swarm optimization (PSO) model and the independent of multi-layer perceptron (MLP) model to evaluate the prediction precision of MLP based on quantum-behaved particle swarm optimization (MLP- QPSO) model. The results show that the hybrid MLP-QPSO model is an optimal prediction tool for estimating daily evaporation, better than the hybrid MLP-PSO model and standalone model. The 400 mm annual precipitation line serves as a natural divide between China’s semi-humid and semi-arid regions, marking a biome separator between forest and grassland vegetation, as well as China’s regions for crop and livestock agriculture. In this study, in order to understand the spatial and temporal patterns of NPP in response to climate change within the fluctuation zone along the 400 mm annual precipitation line, dispersion degree of the annual mean NPP and the variation in the trends of NPP was analyzed by using the coefficient of variation and the unitary linear regression method. The significance of the variation in trends was verified. Previous studies have found that the main driving factors of NPP are temperature and precipitation [29,30]. In this study, the second-order partial correlation analysis method was used to analyze the partial correlation between NPP and precipitation by limiting the influence of evapotranspiration (ET) and land surface temperature (LST). This can effectively avoid the interference of other variables on the analysis results and increase the degree of fitting between the analysis results and actual situation. Furthermore, the effects of ET and precipitation were limited, and the partial correlation between LST and NPP was analyzed. Finally, the Hurst index estimation method was used to predict future trends of NPP in the study area at both annual and seasonal scales. This study reveals the driving forces and limitations of climate factors’ effects on NPP in China’s ecologically sensitive areas and provides a scientific basis for formulating regional environmental governance and protection.

## 2. Materials and Methods

### 2.1. Study Area and Data Sources

**Figure 1:**
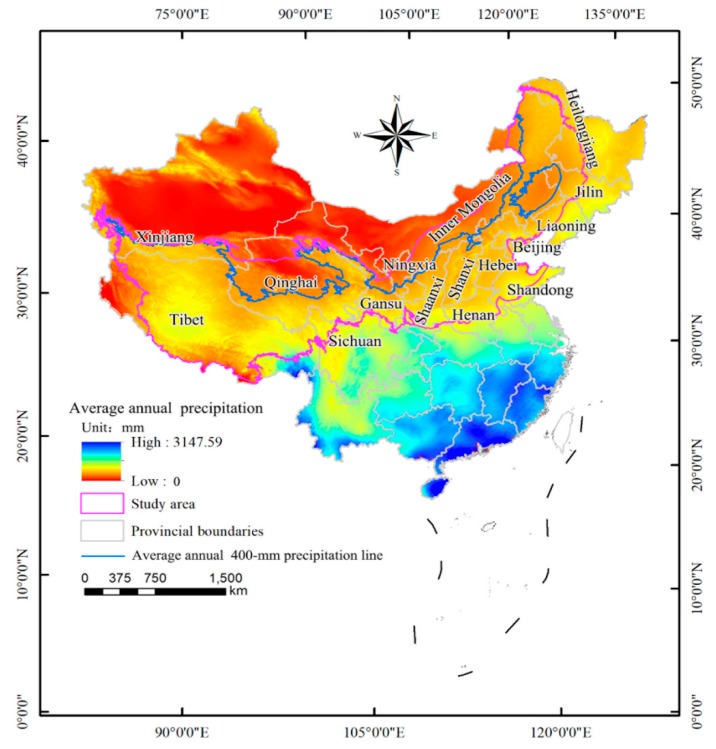
Geographical location of the study area.

The ecotone between agriculture and animal husbandry is bound by a 400 mm precipitation line, but the precipitation is largely random and sudden, resulting in annual differences in the location of the 400 mm precipitation line. Therefore, on the basis of the precipitation data collected since 1951 by 2474 meteorological stations in Mainland China, this study identified the 400 mm annual precipitation line in each year of the study period and took the fluctuation area of the 400 mm annual precipitation line in each year as the study area. Professional meteorological data interpolation software, ANUSPLIN, was used to interpolate the annual precipitation data from weather stations from 1951 to 2015. The interpolated raster of annual precipitation data was used to derive the annual 400 mm precipitation lines for each year. The research area was obtained from the fluctuation range of the 400 mm precipitation line (Figure 1). The study area extends to 303,104 km^2^ and transects 17 provinces, including Heilongjiang, Jilin, Liaoning, Inner Mongolia, Shanxi, Shaanxi, Ningxia, Beijing, Hebei, Henan, Shandong, Qinghai, Gansu, Xinjiang, and Tibet. These provinces span the eastern monsoon, northwest arid, and semi-arid regions and the Tibetan Plateau. The unique geographical location and poor climate conditions of the study area lead to an ecologically sensitive environment that is extremely vulnerable to climate change and anthropogenic activities. The occurrence of frequent droughts, the erosion of soils, desertification, and the loss of biodiversity have been more severe in recent years [31].

NPP data with the temporal resolution of monthly from 2000 to 2015 were extracted from the Moderate-Resolution Imaging Spectroradiometer (MODIS) MOD17A3 dataset and based on the Biome Bio Geochemical Cycles Model (BIOME-BGC), the improved Carnegie Ames Stanford Approach (CASA), and the corrected influence of clouds and aerosols from MODIS LAI-FPAR [16]. This dataset was published by the Numerical Terra Dynamic Simulation Group, University of Montana (http://www.ntsg.umt.edu/ project/mod17). To meet the needs of this study, we used the statistical analysis tool in ArcGIS software (Environmental Systems Research Institute, Redlands, USA) to convert the temporal resolution of NPP data from monthly to seasons and annual. The Land Surface Temperature (LST) dataset with the temporal resolution of annual were provided by the International Scientific & Technical Data Mirror Site, Computer Network Information Center, Chinese Academy of Sciences (http://www.gscloud.cn). The MODIS MOD16A2/ET data with the temporal resolution of annual were provided by the National Aeronautics and Space Administration (NASA) and the Numerical Terra Dynamic Simulation Group (http://www.ntsg.umt.edu/project/ modis/). Mu et al. [32] used the MODIS daily average meteorological reanalysis data and 8-day remote sensing vegetation attributes as inputs to improve the ET algorithm. Their improved algorithm was then used to calculate the net radiation of vegetation and soil by comprehensively considering information such as vegetation coverage, albedo, air temperature, air pressure, and relative humidity. Lastly, the Penman–Monteith model [33] was used to calculate the grayscale remote sensing image dataset of global land surface evapotranspiration. The meteorological monitoring data of all meteorological stations in China from 1951 to 2015 were obtained from the Meteorological Data Sharing Network (http://www.nmic.cn/). From 1951 to 2015, the temporal resolution of the meteorological stations’ precipitation data is monthly. We obtain yearly precipitation data from the accumulated value of the monthly precipitation in each year. Land use data from 2000 to 2015 were obtained from the Resource and Environmental Data Cloud Platform (http://www.resdc.cn). Land use types in this study area were grouped into seven classes: farmland, forest, grassland, water, desert, built-up area, and bare land.

### 2.2. Methods

(1) Spatial Heterogeneity

Global autocorrelation can reflect the similarity degree among spatial neighborhood units. It is used to analyze the distribution pattern and trend of a certain attribute in the whole region. The calculation formula is as follows:

(1) $$I=\frac{n\left(x_{i}-\bar{x}\right)\sum_{i=1}^{n}\sum_{j,j\neq 1}^{n}w_{ij}\left(x_{i}-\bar{x}\right)\left(x_{j}-\bar{x}\right)}{\sum_{i=1}^{n}{\left(x_{i}-\bar{x}\right)}^{2}\sum_{i=1}^{n}\sum_{j=1}^{n}w_{ij}}$$

In this equation, *I* is Moran’s *I*, *n* is the number of observed values, $x_{i}$ and $x_{j}$ are the attribute values of the observations of the regional units *i* and *j*, $\bar{x}$ is the average of the observations, and $w_{ij}$ is the element of the space weight matrix.

Local indicators of spatial association (LISA) is the expression coefficient of local Moran’s *I*, and the calculation formula is:

(2) $$I_{i}=\frac{n\left(x_{i}-\bar{x}\right)\sum_{j,j\neq 1}^{n}w_{ij}\left(x_{j}-\bar{x}\right)}{\sum_{i=1}^{n}{\left(x_{i}-\bar{x}\right)}^{2}}$$

where $I_{i}$ is the local Moran’s I of the ith county-level administrative unit: if $I_{i}$ is a positive value, it indicates that the spatial unit is similar to adjacent units for an attribute; if $I_{i}$ is a negative value, it indicates that the spatial unit is different from the adjacent units for an attribute.

(2) Coefficient of Variation (CV)

The coefficient of variation (CV) is a statistical value that measures the degree to which observed data are dispersed. In this study, the standard deviation in the coefficient of variation calculation reflects the dispersion degree of the annual mean NPP, and the difference in surface vegetation types can be eliminated by dividing by the annual mean. It can be used to not only judge the temporal stability of NPP over the years but also ensure the comparability of temporal stability at the pixel level. Therefore, the CV is a suitable method for analyzing the variation in geographical phenomena and is widely used for the stability analysis of an NPP time series [34]. The CV is a statistical parameter that reflects the degree of variation in NPP, namely, the ratio of the standard deviation to the mean:

(3) $$CV=\frac{SD_{NPP}}{\bar{NPP}}$$

where $SD_{NPP}$ is the standard deviation of NPP, and $\bar{NPP}$ is the average value of NPP. The larger the value of the CV, the larger the fluctuation in NPP. In contrast, a small CV value reflects an NPP time series of vegetation that is relatively stable.

(3) Trend analysis

Unitary linear regression analysis can simulate the variation trend of each grid. In this method, the average annual slope of NPP is fitted by least squares pixel by pixel over a certain period to comprehensively reflect the spatial–temporal evolution characteristics of NPP [35].

(4) $$\mathrm{Slope}=\frac{n\times \sum_{i=1}^{n}i\times P_{i}-\sum_{i=1}^{n}i\sum_{i=1}^{n}p_{i}}{n\times \sum_{i=1}^{n}i^{2}-{\left(\sum_{i=1}^{n}i\right)}^{2}}$$

where Slope is the change in the trend, $P_{i}$ is the NPP for the ith year, and *n* is the length of the time series. If Slope > 0, then NPP has an upward trend. Conversely, NPP has a downward trend if Slope < 0. The significance of the interannual change in NPP is determined by the F test:

(5) $$F=\frac{R^{2}}{1-R^{2}}\times \frac{t-k-1}{k}$$

where *R* is the slope, *t* is the sample number, and *k* is the number of independent variables. The following formula is introduced in this study in order to evaluate the overall change in NPP over a long time series. It is expressed as the relative inter-annual rate of change (RARC_c_) in NPP and obtained by the following formula:

(6) $$RARC_{c}=\frac{Slope}{\bar{NPP}}\times 100\%$$

(4) Second-order partial correlation method

A geographical system is complex and composed of many variables. A change in any element within the system will inevitably affect other elements. The second-order partial correlation analysis method can exclude the effect of other variables and analyze the correlation between the target variables [36]. Thus, the second-order partial correlation analysis can be used to eliminate the interference of other variables to determine the correlation between the remaining two variables. In this study, LST and ET were taken as control variables to analyze the correlation between NPP and precipitation. The correlation between NPP and LST was analyzed by controlling for precipitation and ET. The second-order partial correlation coefficient is calculated from the first-order partial correlation coefficient [37]. The correlation coefficient is calculated by the following formula:

(7) $$r_{xy}=\frac{\sum_{i=1}^{n}\left[\left(x_{i}-\bar{x}\right)\left(y_{i}-\bar{y}\right)\right]}{\sqrt{\sum_{i=1}^{n}{\left(x_{i}-\bar{x}\right)}^{2}\sum_{i=1}^{n}{\left(y_{i}-\bar{y}\right)}^{2}}}$$

Then, the first-order partial correlation coefficient is calculated by the following formula:

(8) $$r_{xy\cdot 1}=\frac{r_{xy}-r_{x1}r_{y1}}{\sqrt{1-r_{x1}^{2}}\sqrt{1-r_{y1}^{2}}}$$

Finally, the second-order partial correlation coefficient is determined by the following formula:

(9) $$r_{xy\cdot 12}=\frac{r_{xy\cdot 1}-r_{x2\cdot 1}r_{y2\cdot 1}}{\sqrt{1-r_{x2\cdot 1}^{2}}\sqrt{1-r_{y2\cdot 1}^{2}}}$$

where 1 and 2 are the control variables; $r_{xy\cdot 12}$ is the partial correlation coefficient of *x* and *y* excluding variables 1 and 2; $r_{x2\cdot 1},r_{y2\cdot 1}$ is the first-order partial correlation coefficient of *x*, *y* and *x*, 2 after excluding 1.

The T-test is used to test the significance of the partial correlation coefficient and is expressed by the following formula [19]:

(10) $$T=\frac{r\sqrt{n-q-1}}{\sqrt{1-r^{2}}}$$

where *r* is the partial correlation coefficient, *n* is the sample number, and q is the number of independent variables.

(5) Hurst index

The Hurst index is an effective method to quantify the long-term dependence of time series information and is widely used in hydrology, economics, climatology, geology, and geochemistry [38,39]. This study applied this method to predict the persistence of NPP trends into the future. The calculation steps are as follows:

(11) $${\bar{NPP}}_{\left(\tau \right)}=\frac{1}{\tau }\overset{\tau }{\underset{t=1}{\sum }}NPP_{\left(\tau \right)}\;\tau =1,2,3,\ldots ,n$$

(12) $$X_{\left(t,\;\tau \right)}=\overset{t}{\underset{t=1}{\sum }}\left({\mathrm{NPP}}_{\left(t\right)}-{\bar{\mathrm{NPP}}}_{\left(\tau \right)}\right)\;1\leq \;t\leq \tau$$

(13) R(τ)=X(t,τ)1≤t≤τmax−X(t,τ)1≤t≤τmin τ=1,2,3,…,n

(14) $$S_{\left(\tau \right)}={\left[\frac{1}{\tau }\overset{\tau }{\underset{t=1}{\sum }}{\left(NPP_{\left(t\right)}-NPP_{\tau }\right)}^{2}\right]}^{\frac{1}{2}}\;\tau =1,2,3,\ldots ,n$$

(15) $$\frac{R_{\left(\tau \right)}}{S_{\left(\tau \right)}}={\left(c\tau \right)}^{H}$$

where c is constant, the H value can be obtained by linear fitting with the least squares method to fit the equation $\log{\left(R/S\right)}_{n}=a+H*\log\left(n\right)$. 0 < H < 0.5 indicates that future changes in the time series will lead to a trend that is opposite of that in the past; 0.5 < *H* < 1 indicates that the time series will remain consistent with the past trend; *H* = 0.5 indicates a random event.

## 3. Results and Discussion

### 3.1. Spatial Heterogeneity Analysis

**Figure 2:**
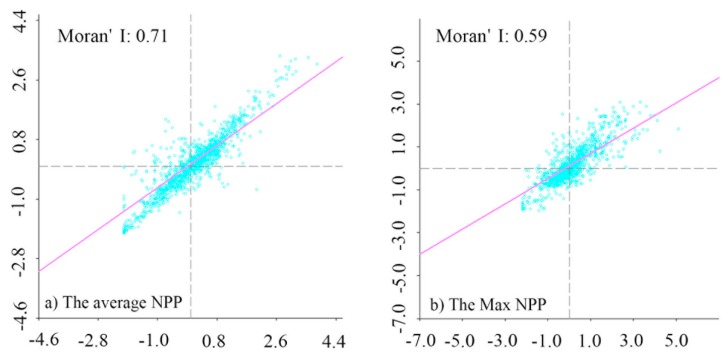
Scatterplot of mean value (**a**) and maximum value (**b**) of NPP in county administrative units.

In this study, the spatial heterogeneity characteristics of NPP were analyzed from global and local scale perspectives. Figure 2 is the scatter diagram of the mean and maximum NPP values in each county-level administrative unit. The Moran’s I index of the mean NPP value and maximum NPP value was 0.71 and 0.59, and the Z values were 40.55 and 35.53, respectively, both of which passed the 1% confidence test. These results show that the global correlation of the mean NPP value was greater than the maximum NPP value. Most county-level administrative units are located in the first and third quadrants of the diagram, and the NPP in the study area had a significant positive correlation on the global scale, with strong spatial clustering and heterogeneity.

**Figure 3:**
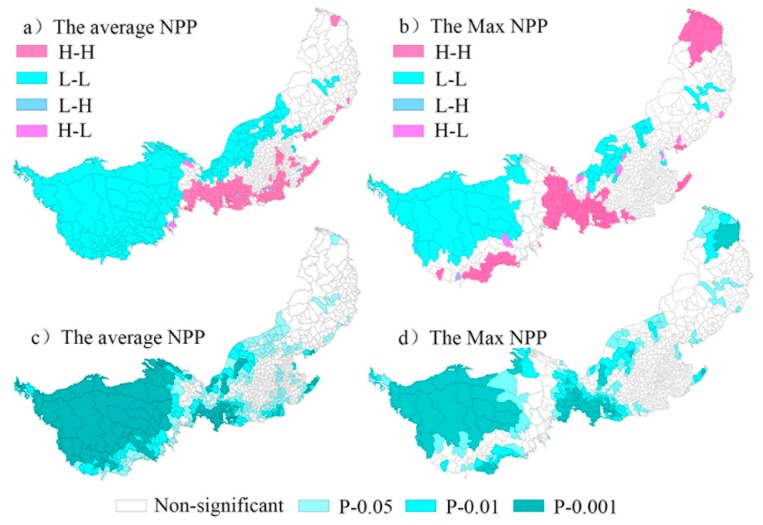
Local spatial agglomeration (**a**,**b**) and significance (**c**,**d**) of average and Max NPP values.

The local autocorrelation results of the mean NPP value show (Figure 3) that the number of county-level administrative units with high-high (HH) and low-low (LL) types accounted for 23.34% of the total number in the study area. There were 10 county-level administrative units with the HL type and 22 with the LH type, accounting for 0.72% and 1.59% of the total, respectively. The number of counties with non-significant results accounted for 51.88% of the total. Combined with Figure 3a, the HH type was mainly distributed in the southeast and eastern marginal areas of the study area. The LL type was mainly distributed in the northwest and west of the study area. The regions that passed the 0.1% significance test were clustered in the northwest of the study area, while regions that passed the 1% significance test were clustered in the southeast of the study area (Figure 3c). The local autocorrelation analysis results of the maximum NPP value show that the number of HH types and LL types accounted for 16.55% of the total number in the study area. There were 18 HL types and 21 LH types with abnormal spatial distribution, accounting for 1.30% and 1.52% of the total, respectively. The number of counties with a non-significant result accounted for 68.79% of the total. Combined with Figure 3b, the HH type was mainly distributed in the southwest, southeast, and northeast of the study area, while the LL type was mainly distributed in the northwest. The regions that passed the 0.1% significance test were clustered in the northwest and northeast of the study area, while the regions that passed the 1% significance test were clustered in the north-central and northern parts of the study area (Figure 3d).

### 3.2. NPP Spatial Pattern and Land Use Change

**Figure 4:**
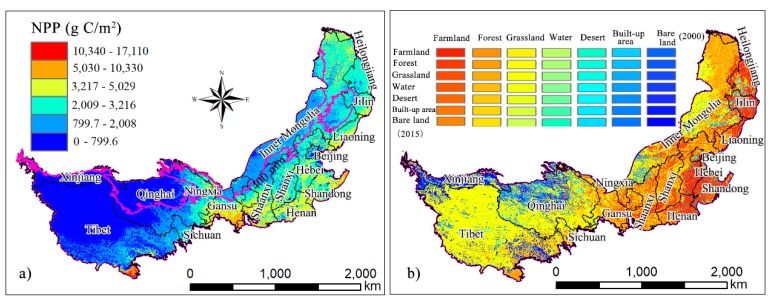
Mean annual NPP from 2000 to 2015 (**a**) and the spatial pattern of conversion of land use types from 2000 to 2015 (**b**).

The spatial pattern of NPP in the study area for the 2000–2015 period was obtained by using pixel-level statistics and was divided into seven grades by the natural discontinuity method (Figure 4a). The variable spatial distribution of NPP is consistent with the distribution of precipitation, with a gradual decrease from the southeast to the northwest. This is because, except for some parts of the Tibetan Plateau, most regions of China have less precipitation, resulting in a lack of moisture for vegetation growth. Under the background of climate warming, soil moisture evaporation in arid and semiarid areas in northwest China intensifies the degree of drought. Thus, precipitation is an important limiting factor for NPP in areas with a low moisture. In addition, the surface elevation of the study area gradually increases from east to west, making it difficult for the warm and humid airflow in the southeast coast to reach the northwest inland region. On the other hand, the rise of elevation causes a decrease in temperature, further limiting the growth of crops. This phenomenon is most obvious on the Tibetan Plateau. The average NPP in the research area was 1640.19 g C/m^2^ during the study period. Generally, the average NPP in the east and south were higher than that in the west and north, respectively. Areas in which the NPP was in the range of 800–2008 g C/m^2^ were concentrated in Inner Mongolia and the southeastern edge of the Tibetan Plateau. This accounted for 22.28% of the total study area. The NPP in the southeastern part of the study area ranged from 3217 to 10330 g C/m^2^, accounting for 15.19% of the study area. This area has relatively good hydrothermal conditions and includes southern Gansu and Shaanxi, most of Henan, Shandong, and central Hebei. The NPP in the Tibetan Plateau was significantly lower than that in other regions, and only the southeast region had an NPP that was slightly higher (800–3216 g C/m^2^), while the NPP in most other regions was lower than 800 g C/m^2^. Precipitation in the southeastern region of the study area was higher as a result of the warm and humid airflow from the Indian Ocean that gets uplifted by the terrain, while the northwest of the Tibetan Plateau, located deep inland and at a higher altitude, had less precipitation and lower temperatures. The part of the study area in which the spatial distribution of NPP was 2008 g C/m^2^ correlated with an annual average precipitation of 400 mm. According to the spatial patterns of the land use transfer matrix for 2000–2015 (Figure 4b), farmland was mainly found in the Henan, Shandong, Hebei, Shaanxi, Shaanxi, Jilin, and Liaoning provinces, which have good hydrothermal and topographic conditions. Grassland and bare land were predominantly found in Qinghai, Xinjiang, and Inner Mongolia, and the distribution of NPP corresponded to the land use type.

Table 1 shows the statistical results of land use transfers that occurred from 2000 to 2015. According to the retention rate of land use types, from 2000 to 2015, the study area was dominated by grassland (179.35 × 10^4^ km^2^) and farmland (66.0 × 10^4^ km^2^), accounting for 48.29% and 17.77% of the total study area, respectively. Forest was the third most dominant land use type (46.26 × 10^4^ km^2^) and accounted for 12.46% of the study area. The desert areas were the smallest (6.94 × 10^4^ km^2^). The area of farmland converted into desert was the largest of the land use transfer areas, reaching 8956 km^2^. The area of grassland that was converted into desert was 2648 km^2^. This indicates that the study area is ecologically sensitive and at risk of further deterioration. The area converted from grassland to farmland was 4994 km^2^ and from farmland to grassland was 4346 km^2^. There was a dramatic mutual transformation process between the two land types, indicating that land use in the study area has been significantly affected by human activities. In addition, 2856 km^2^ of built-up area was transferred to grassland. This may be closely related to the Chinese government’s strict control of 120 million hectares of arable land in recent years and its resolute clampdown on the illegal occupation of arable land. The area of grassland that was converted into built-up area was 3418 km^2^, reflecting the blind pursuit of rapid expansion through urbanization in recent years, even at the cost of the environment.

### 3.3. CV and Trend of NPP

**Figure 5:**
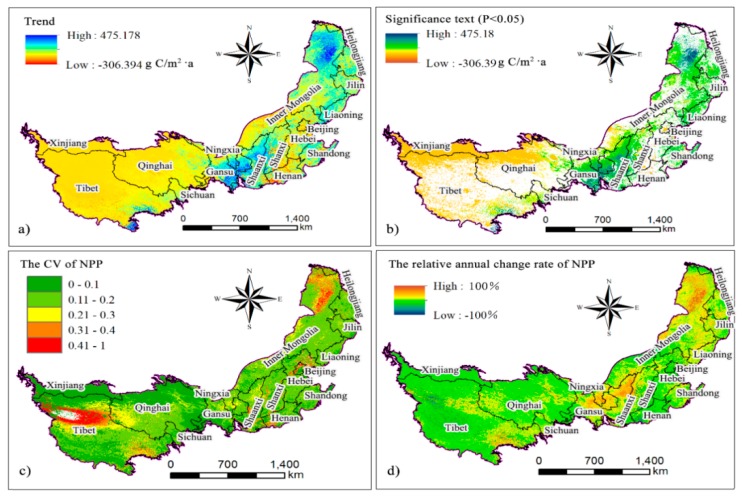
The NPP variation trends (**a**), F significance test (**b**), CV of NPP (**c**), and relative annual rate of change in NPP (**d**).

From 2000 to 2015, the average annual variation in NPP was 20.57 g C/m^2^·a (Figure 5a), indicating that the NPP as a whole trended upward during the research period. The maximum rate of growth in the inter-annual variation in NPP was 475.18 g C/m^2^·a, which increased significantly in the eastern Gansu, North-central Shaanxi, and northeastern Inner Mongolia regions. The maximum annual rate of NPP decline was 306.39g C/m^2^·a. The NPP in Henan, Shanxi, Hebei, and central Inner Mongolia had a significant downward trend, while that in Qinghai, Tibet, Xinjiang, and western Inner Mongolia had a slight downward trend. The significance test results of the inter-annual changes in NPP (Figure 5b) show that NPP increased significantly. The regions in the northeastern part of the study area that passed the significance test (*p* < 0.05) were mainly distributed in Inner Mongolia and the Jilin and Liaoning provinces; in the middle of the study area, north-central Shaanxi, central and western Shanxi, southern Ningxia, and eastern Gansu provinces passed the significance test (*p* < 0.05). The main reason for this result is the construction of the Grain for Green Program and the Three Northern Shelter Forest Programs over the last 35 years [40], effectively restoring the vegetation in the above-mentioned areas. The global temperature is increasing, and China’s climate has also changed significantly in the past 100 years, consistent with the general trend of global temperature change [41]. The vegetation activity in most parts of China is also increasing due to earlier or longer growing seasons caused by global warming [42]. In addition, an increase in people’s awareness of environmental protection and rationalization and scientization of agricultural planting both significantly affect the upward trend of NPP. The NPP in the southern part of Ningxia increased significantly, and the rate of increase dropped from the south to the north. This is consistent with the conclusion reached by Zhu et al. [18]. The significant increase in NPP in north-central Shaanxi in the central part of the study area is consistent with the conclusion reached by Li et al. [43]. In addition, NPP in the northeastern part of the study area had an upward trend because of the widespread forest in this area, a small human population, and minimal anthropogenic influence. The water in the area is supplied by precipitation, snow cover, and meltwater from permafrost [44]. The regions in which NPP decreased significantly and passed the significance test (*p* < 0.05) were mainly distributed in the Tibetan Plateau (Qinghai, Tibet, and Xinjiang). The observed decreasing trend for NPP in the Tibetan Plateau is consistent with the conclusion reached by Han et al. [45]. The coefficient of variation of the NPP in the study area was between 0 and 1.4, with an average CV of 0.144, indicating a small overall variation in NPP during the study period. The regions with larger CV ranges were distributed in the northeastern part of Inner Mongolia and southeastern part of Tibet, with scattered distributions around Beijing, Shaanxi, Shanxi, and Henan (Figure 5c). The NPP trends, the CV (Figure 5d), and the relative annual rate of change showed strong spatial consistencies, indicating that the NPP in the northeast, middle, and southwest regions of the study area changed dramatically from 2000 to 2015.

### 3.4. Influence of Climate on NPP

**Figure 6:**
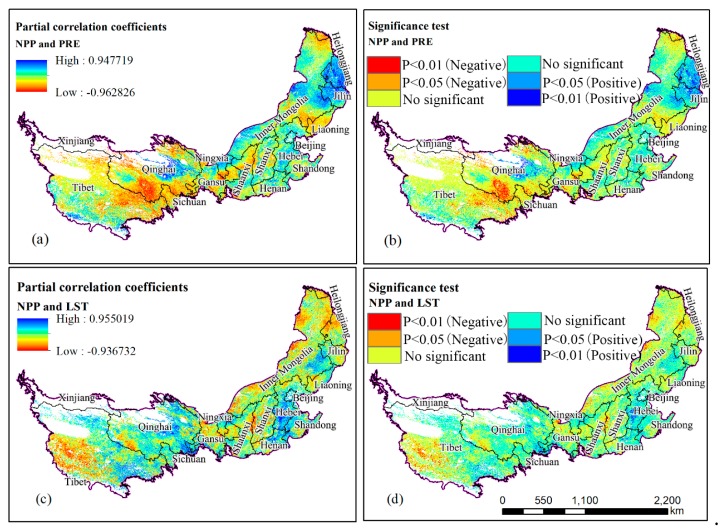
Spatial distribution and the T significance test of partial correlations coefficient between annual NPP and precipitation (**a**,**b**) and NPP and Land surface temperature (**c**,**d**) between 2000 and 2015.

The correlation between NPP and the climate was the main focus of previous research [46,47]. This study focused on analyzing the influence of climate factors on NPP (Figure 6a–d). To exclude the interference of other variables on NPP, this study adopted the second-order partial correlation analysis method to analyze the correlation between NPP and precipitation and LST (Figure 6a,c). Firstly, the ET and LST were used as control variables to analyze the partial correlation between NPP and the target variable (precipitation). Secondly, precipitation and EP were used as control variables to analyze the partial correlation between NPP and target variable (LST). Table 2 shows the statistical results of the second-order partial correlation between NPP, precipitation, and LST for the 17 provinces. A significance test was used to determine the correlation (Figure 6b,d).

The partial correlation coefficient between NPP and precipitation was between −0.96 and 0.95, and the positive and negative correlation areas accounted for 39.24% and 60.76% of the study area, respectively (Table 2). This indicates that there was a negative correlation between NPP and precipitation in the study area. The regions with a positive correlation between NPP and precipitation were concentrated in eastern and western Inner Mongolia, western Jilin, and Liaoning, as well as most of Hebei and central Qinghai, which have experienced severe water shortage and are distant from the ocean. The abovementioned areas are severely affected by drought due to the lack of precipitation and high rate of evaporation. Relevant studies show that the trend of warming and drying in the inland areas of northwest China, especially the Loess Plateau, is strengthening [48]. An upward trend of temperature promoted an increase in soil moisture evaporation, further aggravating water shortage in these regions. Therefore, water resources are the main limiting factor for vegetation growth. This is why NPP and precipitation have a positive correlation in arid and semiarid regions. This positive correlation between NPP and precipitation in the arid and rainless steppe region of Inner Mongolia is consistent with the conclusion obtained by Pan et al. [49] through a correlation analysis between NPP and meteorological factors that were based on data measured over the last 30 years in the Hulinbeier forest and grassland transition zone. The negative correlation was concentrated on the border between Qinghai and Tibet, the northeastern part of Inner Mongolia, and the border between Inner Mongolia and Liaoning. Water resources in the above areas, particularly the southeastern edge of Tibetan Plateau, are relatively abundant. Precipitation decreases at higher altitude. This, in turn, reduces regional temperatures and limits the NPP. The partial correlation coefficient of NPP and precipitation passed the significance test (*p* < 0.01) over an area of 11.12 × 10^4^ km^2^, accounting for 3.67% of the study area. The area with a positive correlation for 1.39% of the total and was mainly distributed in Jilin and Liaoning. Regions with a negative correlation accounted for 2.28% of the total area, mainly distributed at the Qinghai and Tibet border. This is consistent with the conclusion that the NPP was negatively correlated with temperature, precipitation, and solar radiation in relevant studies [50].

The partial correlation coefficient between NPP and LST was between −0.94 and 0.96, and the positive and negative correlation regions accounted for 52.64% and 47.36% of the total study area, respectively (Table 2). The regions with a positive correlation were concentrated in Hebei, Henan, Shandong, Qinghai, and Tibet, while the regions with a negative correlation were concentrated in Jilin, Liaoning, Inner Mongolia, Shaanxi, and Shanxi. Although temperature was one of the most important climatic conditions for the growth of vegetation, higher surface temperatures accelerated the evaporation of water in the regions with negative correlations between NPP and LST because of the lack of water resources. In addition, NPP and LST were negatively correlated in southwest Tibet. Precipitation, NPP, and LST showed significant positive correlations in Hebei, eastern Inner Mongolia, and western Qinghai. The change in LST and precipitation has bidirectional regulating effect on NPP. An increase in surface temperature promoted the growth of vegetation before reaching the critical point of heat required for photosynthesis. When the rate at which surface temperatures rise exceeds the appropriate threshold of vegetation photosynthesis, it would accelerate the consumption of nutrients and increase the amount of water evaporation of soil. Therefore, the rise of LST played a positive role in promoting the increase of NPP in the southeast of study area with a relatively good soil moisture and the Tibetan plateau. This can be attributed to the relative abundance of water resources in the abovementioned areas. However, NPP played an inhibitory role in the arid and semiarid areas in northwest China. The partial correlation coefficient of NPP and LST passed the significance test (*p* < 0.01) over an area of 7.25 × 10^4^ km^2^, accounting for 2.39% of the study area. The area with a positive correlation accounted for 1.22% of the total, while the area with a negative correlation accounted for 1.17%. Areas with a positive correlation accounted for 4.16% of the study area, while areas with a negative correlation accounted for 4.47%. In general, the NPP in southeastern Tibet was positively correlated with temperature, while NPP was negatively correlated with precipitation. This is because of the abundance of precipitation in the area and the influence of elevation factors, the LST of the Tibetan Plateau is lower all the time; the lower surface temperature is the main factor that limits the vegetation growth. However, the occurrence of precipitation events increases the total cloud cover and soil moisture, leading to a decrease in sunshine hours and solar radiation and decrease in surface temperature, further aggravating the inhibiting effect of low temperature on NPP in the abovementioned regions. Similarly, under the background of climate change, the continuous rise of LST will directly affect the extension of crop growing season. This has a positive co-advancement effect on NPP. This is consistent with previous studies that found a greater influence of surface temperature on NPP than that of precipitation in this region [51,52]. Remote sensing can objectively reflect the influence of natural conditions and human factors on vegetation growth. In future research, the influence of climate factors and human activities on regional NPP should be studied individually to analyze the internal driving mechanism of NPP evolution from a more detailed perspective.

### 3.5. Persistence Analysis of NPP Variation

**Figure 7:**
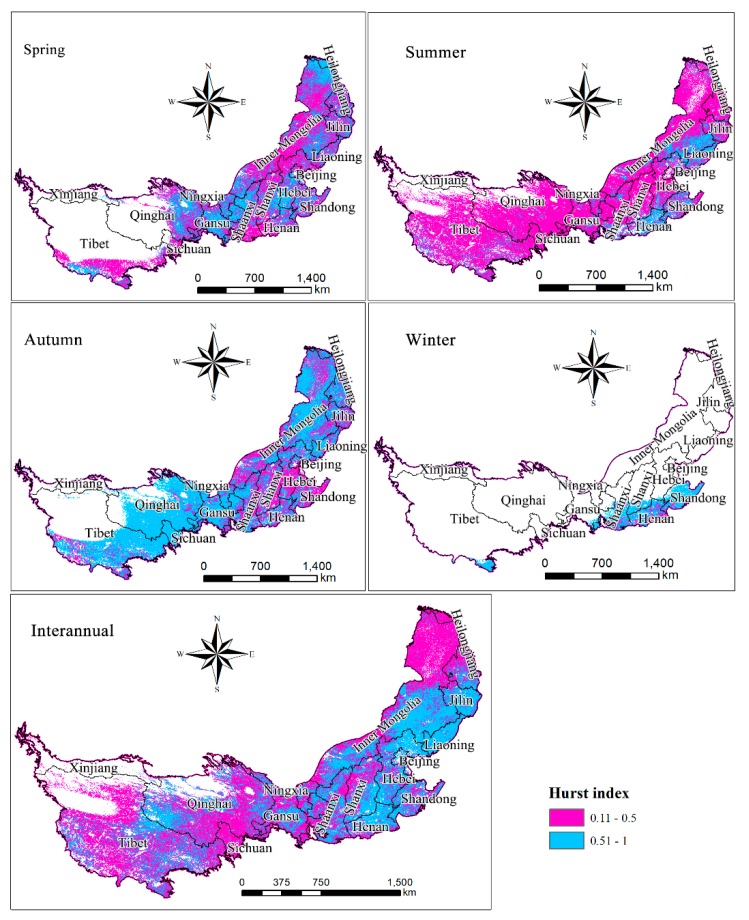
Future trend analysis of net primary productivity in the study area.

This study was based on the use of annual and seasonal NPP raster data from 2000 to 2015. The Hurst index estimation method was used to interpolate the future trends of NPP. The persistent changes in NPP showed significant differences on different time scales for future predictions (Figure 7). During spring, the NPP persisted over 95.38 × 10^4^ km^2^ (25.67% of the study area). This was mainly distributed in Qinghai, Eastern Gansu, Ningxia, northeastern Inner Mongolia, and the eastern/central parts of Hebei and Shanxi. An area of 49.78 × 10^4^ km^2^ (13.40%) showed continued persistence of NPP in the summer. NPP showed continued persistence into the future and was mainly distributed in two regions: (1) Henan and Shandong and (2) the border between Inner Mongolia and Liaoning. In autumn, NPP persisted over an area of 185.52 × 10^4^ km^2^ (49.92%) and was mainly distributed in Qinghai, southeastern Tibet, eastern Gansu, western Ningxia and Shaanxi, and most of northeastern Inner Mongolia. The NPP persisted over an area of 25.09 × 10^4^ km^2^ (6.75% of the total study area) during winter. This was mainly distributed in Shandong, northern Henan, southern Shaanxi, and northern Shanxi. Additionally, there were a few areas in the southeastern corner of Tibet in which the NPP was more persistent. On an annual scale, the Hurst index estimate was between 0.11 and 1, with a mean of 0.497 and a standard deviation of 0.10. Fifty-four percent (159.54 × 10^4^ km^2^) of the study area had a Hurst index of <0.5, indicating that the NPP will persist into the future. The regions with a Hurst index of >0.5 were mainly distributed in the eastern provinces that have good hydrothermal conditions; these regions include Inner Mongolia, northern Shaanxi, the border of Liaoning and Hebei, and the border of Shanxi, Henan, and Hebei. The NPP in the eastern part of Inner Mongolia and the northern part of Shaanxi showed significant persistence, consistent with the findings of Tong et al. [53], who interpolated future trends of persistence related to the vegetation growth in the Mongolian Plateau. The strong persistence of NPP around Beijing, Tianjin, and Hebei indicates that the vegetation in these areas continuously improved [48,54]. Additionally, the southern and northern parts of Ningxia also showed a trend of persistence of NPP. This is consistent with the conclusion by Zhao et al. [55].

## 4. Conclusions

Analyses of NPP trends, calculations of the coefficient of variation, and the second-order partial correlation coefficient were used to study the spatial and temporal changes and interpolate future trends of NPP. Climate factors as a driver of NPP are discussed. The following conclusions are drawn.

First, the spatial distribution of NPP was consistent with that of precipitation and showed obvious spatial heterogeneity. The spatial distribution for which the NPP of the study area was 2008 g C/m^2^ is consistent with an annual average precipitation of 400 mm. Grassland and farmland accounted for 179.35 × 10^4^ km^2^ (48.29%) and 66.0 × 10^4^ km^2^ (17.77%) of the study area. The average annual variation tendency of NPP was 20.57 g C/m^2^·a, indicating that the NPP in the study area had an overall upward trend from 2000 to 2015. The maximum rate of increase in NPP was 475.18 g C/m^2^·a, and the maximum rate of decrease was 306.39 g C/m^2^·a. The upward trend of NPP in the study area from 2000 to 2015 may be closely related to the large-scale implementation of the Grain for Green Program and the Three Northern Shelter Forest Programs that have been run by the Chinese government since 1999. The rapid development of agricultural technology may also have played a role in the upward trend of NPP.

Second, the second-order partial correlation coefficient of NPP and precipitation was between −0.96 and 0.95. The areas with positive and negative correlations accounted for 39.24% and 60.76% of the total study area, respectively. The area for which the partial correlation coefficient passed the significance test (*p* < 0.01) was 11.13 × 10^4^ km^2^, accounting for 3.67% of the total area. The partial correlation coefficient between NPP and LST was between −0.94 and 0.96, and the areas with positive and negative correlations accounted for 52.64% and 47.36% of the total area, respectively. The area for which the partial correlation coefficient passed the significance test (*p* < 0.01) was 7.25 × 10^4^ km^2^ (2.39% of the total study area). On the whole, NPP in the study area shows a negative correlation with precipitation and a positive correlation with temperature, which may be because the study area contains a large portion of the Tibetan Plateau, which predominately has year-round cold, wet conditions. The perennial low temperature condition in the Tibetan Plateau is the limiting factor for crop growth, and the occurrence of precipitation events will increase the cloud and soil moisture, thus aggravating the negative effects of low temperatures.

Finally, the persistence of NPP showed significant differences on different time and seasonal scales. The biggest persistence of NPP was observed for autumn (185.52 × 10^4^ km^2^ or 49.92% of the study area), followed by spring and summer, accounting for 25.67% and 13.40% of the total area, respectively. The lowest persistence of NPP was in winter, in which only 25.09 × 10^4^ km^2^ (6.75%) of the study area showed persistence of NPP. On an annual scale, the region with a Hurst index of >0.5 accounted for 46% of the total study area and was distributed in the eastern part of the study area. The NPP shows obvious anti-persistence characteristics on the annual scale, indicating that although NPP in the research area has increased over the past 20 years, NPP is likely to be degraded in the future due to the strong ecological vulnerability and the complex, changing climatic conditions.

In this study, when analyzing the effect of climate factors on NPP change, only the temperature, precipitation, and evaporation that have the most obvious effect on NPP were introduced. However, in fact, vegetation change is the result of combined effect of many climatic factors, such as temperature, precipitation, soil relative humidity, soil texture, solar radiation, and terrain. Therefore, when quantifying the effect of climate change on vegetation, the comprehensive effect of various factors on vegetation should be considered to more comprehensively and objectively quantify the internal driving mechanism of NPP change. The coupling relationship between vegetation and climate change is a complex system. The stability of different vegetation types and their adaptability to climate change differ from each other. Therefore, further research should focus on the effect of climate change on different types of land use. Human activities have both positive and negative effects on NPP changes. The residual analysis method should be used to evaluate the positive and negative effects of human activities on NPP change and analyze the contribution rates of climate change and human activities to NPP.

## Figures and Tables

**Table 1:** Land use transfer matrix from 2000 to 2015 (km^2^).

Land Use Type	Farmland	Forest	Grassland	Water	Desert	Built-Up Area	Bare Land
Farmland	659,998	2360	4346	1405	8956	124	294
Forest	764	46,2587	890	167	468	27	46
Grassland	4994	2966	1,793,473	1758	2648	3418	322
Water	1803	138	899	16,4937	466	1036	244
Desert	181	27	57	82	69,398	14	1
Built-up area	747	302	2856	1513	744	250,059	155
Bare land	18	7	101	73	15	11	265,807

**Table 2:** The statistical results of the second-order partial correlation between NPP, precipitation, and LST.

Province	NPP and Precipitation (R)			NPP and Precipitation (Area, 10^4^km^2^)		NPP and LST (R)			NPP and LST (Area, 10^4^km^2^)	
	Max	Min	Mean	Positive	Negative	Max	Min	Mean	Positive	Negative
Heilongjiang	0.67	−0.84	−0.19	0.32	0.69	0.90	−0.57	0.23	0.84	0.17
Jilin	0.96	−0.96	−0.15	20.18	52.09	0.97	−0.94	0.01	38.51	33.74
Liaoning	0.66	−0.87	−0.33	0.37	5.37	0.91	−0.70	0.19	4.58	1.16
Hebei	0.74	−0.88	−0.12	5.04	11.29	0.87	−0.90	0.00	8.38	7.95
Beijing	0.83	−0.81	0.00	6.77	7.44	0.89	−0.86	−0.09	5.07	9.14
Tianjin	0.81	−0.88	−0.11	2.71	6.88	0.86	−0.77	0.15	6.96	2.61
Shandong	0.94	−0.94	−0.20	12.31	36.04	0.91	−0.89	0.12	33.27	15.08
Henan	0.82	−0.82	0.09	2.76	1.25	0.87	−0.79	0.08	2.47	1.54
Shanxi	0.90	−0.95	0.02	34.07	31.74	0.92	−0.94	−0.11	21.27	44.50
Shaanxi	0.89	−0.77	−0.27	0.35	5.93	0.89	−0.83	0.11	4.40	1.87
Inner Mongolia	0.90	−0.60	0.34	7.13	0.56	0.86	−0.79	−0.03	3.23	4.47
Ningxia	0.91	−0.89	0.26	8.87	2.25	0.84	−0.86	−0.19	1.84	9.28
Gansu	0.77	−0.84	−0.09	2.69	6.22	0.87	−0.78	0.13	6.12	2.78
Xinjiang	0.73	−0.48	0.13	0.82	0.20	0.83	−0.59	0.24	0.90	0.13
Sichuan	0.81	−0.60	0.12	0.30	0.09	0.80	−0.65	0.16	0.30	0.09
Qinghai	0.80	−0.76	0.03	8.91	6.96	0.93	−0.85	0.17	11.61	4.27
Tibet	0.87	−0.91	−0.18	4.46	14.09	0.90	−0.89	0.00	8.70	9.85
